# Supplementary material for: Genetic Variation in CYP2D6 and SLC22A1 Affects Amlodipine Pharmacokinetics and Safety
Source: Pharmaceutics. 2023 Jan 25;15(2):404. doi: 10.3390/pharmaceutics15020404 (PMC9959242; doi:10.3390/pharmaceutics15020404)
Supplement: Supplementary file 1 [file pharmaceutics-15-00404-s001.zip › pharmaceutics-2173746-supplementary.pdf]

Genetic variation in CYP2D6 and SLC22A1 affects amlodipine pharmacokinetics and safety.

Paula Soria-Chacartegui[1], Pablo Zubiaur[1, 2*], Dolores Ochoa[1], Gonzalo Villapalos-García[1], Manuel Román[1], Miriam Matas[1], Laura Figueiredo-Tor[1], Gina Mejia-Abril[1], Sofía Calleja[1, 3], Alejandro de Miguel[1], Marcos Navares-Gómez[1], Samuel Martín-Vilchez[1], Francisco Abad-Santos[1, 4*].

[1]Clinical Pharmacology Department, Hospital Universitario de La Princesa, Instituto Teófilo Hernando, Universidad Autónoma de Madrid (UAM), Instituto de Investigación Sanitaria La Princesa (IP), Madrid, Spain.

[2]Division of Clinical Pharmacology, Toxicology and Therapeutic Innovation, Children's Mercy Research Institute, Kansas City, MO, United States.

[3]Servicio de Bioquímica Clínica, Clínica Universidad de Navarra, Pamplona, Spain.

[4]Centro de Investigación Biomédica en Red de Enfermedades Hepáticas y Digestivas (CIBERehd), Instituto de Salud Carlos III, Madrid, Spain

*** Correspondence:**

Pablo Zubiaur Precioso, PhD.
Clinical Pharmacology Department,
Hospital Universitario de La Princesa
C/ Diego de León, 62. 28006 Madrid, Spain
Tel: 34 915202425; Fax: 34 915202540.
E-mail: pablo.zubiaur@salud.madrid.org

Francisco Abad Santos, PhD, MD.
Clinical Pharmacology Department,
Hospital Universitario de La Princesa
C/ Diego de León, 62. 28006 Madrid, Spain
Tel: 34 915202425; Fax: 34 915202540
E-mail: francisco.abad@uam.es

Supplementary material

Table S1. Pharmacokinetic characteristics regarding genotypes or phenotypes in the exploratory step.

**Genotype or Phenotype**	**N**	**AUC_{72}/DW (h*ng*kg/ml*mg)**	**C_{max}/DW (ng*kg/ml*mg)**	**t_{max} (h)**	**$t_{1/2}$ (h)**
***ABCB1* rs1128503**					
CC	29	2131.54 (466.17)	65.52 (13.07)	6.19 (1.49)	35.07 (6.15)
CT	24	2104.83 (461.10)	62.57 (11.58)	6.27 (1.44)	35.91 (7.94)
TT	16	2189.45 (362.13)	66.53 (10.77)	5.75 (1.25)	35.36 (4.90)
***ABCB1* rs1045642**					
CC	25	2149.19 (495.14)	64.43 (14.10)	6.18 (1.50)	35.07 (6.19)
CT	26	2156.73 (433.74)	66.02 (10.19)	5.96 (1.17)	36.59 (7.80)
TT	18	2086.51 (372.93)	63.27 (11.75)	6.25 (1.67)	34.25 (4.71)
***ABCC2* rs2273697**					
GG	48	2103.45 (374.26)	64.81 (10.94)	6.13 (1.40)	35.63 (5.73)
GA + AA	20	2224.49 (569.74)	64.91 (14.71)	6.05 (1.52)	35.24 (8.27)
***ABCG2* rs2231142**					
NM	51	2130.59 (454.33)	64.75 (12.20)	6.08 (1.39)	35.76 (7.01)
IM	18	2150.09 (397.45)	64.67 (11.77)	6.22 (1.54)	34.49 (4.85)
***CYP1A2* rs2470890**					
T/T	14	2023.67 (449.10)	60.96 (12.45)	5.89 (1.38)	34.58 (9.99)
T/C	41	2122.74 (437.44)	65.39 (11.09)	6.06 (1.41)	35.69 (5.45)
C/C	14	2285.58 (414.91)	66.55 (14.12)	6.50 (1.51)	35.52 (5.40)
***CYP1A2* rs2069514**					
G/G	56	2134.74 (456.07)	64.66 (12.15)	6.04 (1.49)	35.40 (7.00)
G/A	9	2080.78 (305.59)	63.11 (7.98)	6.50 (1.20)	36.50 (3.37)
A/A	4	2272.35 (490.81)	69.26 (18.84)	6.38 (0.48)	33.39 (4.48)
***CYP1A2* rs762551**					
C/C	30	2031.07 (400.04)	62.21 (11.66)	6.07 (1.47)	34.30 (7.40)
C/A	35	2206.84 (444.21)	66.90 (12.02)	6.07 (1.35)	36.15 (5.69)
A/A	4	2297.62 (591.81)	64.56 (13.91)	6.88 (1.75)	37.54 (6.22)
CYP2A6					
*1/*1	62	2135.29 (435.85)	64.89 (12.49)	6.08 (1.28)	35.41 (6.60)
*1/*9	5	2258.10 (533.73)	63.43 (6.99)	7.3 (2.31)	36.88 (6.72)
*9/*9	2	1841.65 (189.61)	62.79 (7.78)	4.25 (1.06)*	32.26 (3.32)
**CYP2B6**					
RM	18	2008.81 (336.16)	65.39 (11.84)	6.06 (1.32)*[1]	32.29 (4.21)*[2]
NM	20	2256.64 (467.49)	66.95 (11.32)	5.73 (1.59)*[1]	36.93 (5.57)
IM	23	2066.54 (494.47)	60.64 (12.07)	6.39 (1.49)	35.32 (6.94)

PM	6	2321.79 (346.58)	69.55 (14.83)	6.5 (0.45)	39.03 (10.99)
**CYP2C19**					
RM	10	2253.59 (454.23)	65.69 (17.42)	6.75 (1.34)	36.66 (4.68)
NM	37	2120.85 (439.17)	64.57 (12.00)	6.09 (1.50)	35.65 (7.17)
IM	21	2102.90 (449.32)	64.54 (9.66)	5.79 (1.24)	34.54 (6.31)
**CYP2C9**					
NM	44	2154.12 (472.71)	64.78 (13.36)	6.20 (1.37)	36.15 (6.98)
IM	23	2117.12 (384.22)	65.52 (9.27)	5.98 (1.56)	34.31 (5.58)
PM	2	1943.24 (240.09)	54.51 (6.61)	6.00 (1.41)	32.38 (5.27)
**CYP3A5**					
NM	2	2367.01 (439.75)	72.06 (1.69)	5.50 (1.41)	35.20 (5.30)
IM	11	2053.53 (549.79)	59.55 (14.67)	6.41 (2.01)	33.36 (5.73)
PM	56	2143.55 (418.18)	65.48 (11.45)	6.08 (1.30)	35.84 (6.70)
***SLC22A1* rs72552763**					
GAT/GAT	44	2096.33 (423.33)	63.48 (11.31)	5.92 (1.41)	35.03 (6.20)
GAT/-	23	2232.01 (466.44)	67.89 (12.95)	6.30 (1.30)	35.98 (7.34)
-/-	2	1893.50 (345.62)	55.84 (12.02)	8.25 (1.77)	37.77 (4.22)
***SLC22A1* rs12208357**					
C/C	63	2162.95 (438.89)	65.18 (12.17)	6.14 (1.44)	35.58 (6.71)
C/T + T/T	6	1849.32 (325.34)	59.93 (9.66)	5.83 (1.21)	33.85 (3.88)
**SLCO1B1**					
Normal function (NF)	56	2150.83 (436.30)	65.14 (11.85)	6.23 (1.35)	35.92 (6.42)
Decreased function (DF)	11	2025.92 (469.48)	61.57 (13.70)	5.41 (1.73)	32.75 (6.89)
Poor function (PF)	2	2314.94 (357.10)	70.62 (0.36)	6.75 (0.35)	36.26(6.79)
**UGT1A1**					
NM	30	2184.19 (511.07)	65.03 (13.03)	6.37 (1.66)	36.89 (7.58)
IM	35	2074.46 (382.75)	63.27 (10.91)	5.93 (1.22)	34.26 (5.57)
PM	4	2307.48 (212.81)	75.17 (10.18)	5.88 (0.95)	34.67 (3.83)
**CYP2C8**					
RM + UM	15	2182.61 (424.83)	64.46 (10.22)	6.50 (1.21)	35.20 (6.41)
NM	40	2095.00 (429.85)	64.59 (13.61)	6.09 (1.54)	35.47 (6.72)
PM + IM	11	2287.72 (506.18)	67.12 (9.57)	6.14 (0.87)	36.46 (6.88)

No genetic information was available for 1 volunteer, as no sample could be recovered. The total number of volunteers for some genes is lower than 69 due to errors in the genotyping technique.
